# Migraine Headaches: The Predictive Role of Anger and Emotional Intelligence

**DOI:** 10.30476/IJCBNM.2021.90552.1706

**Published:** 2022-01

**Authors:** Maryam Shaygan, Elham Saranjam, Ameneh Faraghi, Zinat Mohebbi

**Affiliations:** 1 Community Based Psychiatric Care Research Center, Department of Psychiatric Nursing, School of Nursing and Midwifery, Shiraz University of Medical Sciences, Shiraz, Iran; 2 Student Research Committee, School of Nursing and Midwifery, Shiraz University of Medical Sciences, Shiraz, Iran; 3 Department of Medical Surgical Nursing, School of Nursing and Midwifery, Shiraz University of Medical Sciences, Shiraz, Iran

**Keywords:** Anger, Emotional intelligence, Migraine

## Abstract

**Background:**

Research has shown that emotional intelligence and anger are significant predictors of both subjective and objective health. The present study aimed to draw a comparison between migraine patients and healthy individuals in terms of emotional intelligence and anger. In addition, there was an attempt to investigate the predictive role of emotional intelligence and anger in chronic migraine.

**Methods:**

This comparative study was carried out on 494 individuals including patients with chronic migraine (n=250) and healthy controls (n=244) in Shiraz between August 2019 and February 2020. The participants with chronic migraine and healthy controls were selected using convenience sampling and multistage sampling, respectively. Participants completed validated self-report questionnaires: Bradberry and Greaves emotional intelligence test and the provocation inventory. The data were analyzed using SPSS software (version 22.0) and chi-square test, t test and logistic regression were used. The significance level was set at P<0.05.

**Results:**

The results of independent t-test indicated that the mean intensity of anger was significantly higher among the patients with migraine (51.52±15.66) compared to the healthy controls (28.39±9.85) (P<0.001). The mean score of emotional intelligence was significantly lower among the patients with migraine (75.92±8.23) in comparison to the healthy controls (116.23±12.28) (P<0.001). Binary logistic regression revealed that neither age (P=0.72), sex (P=0.62), marital status (P=0.63) and education level (P=0.68), nor anger (P=0.24) was significantly associated with chronic migraine. However, emotional intelligence had a negative association with chronic migraine (B=-1.13, OR=0.32, P<0.001).

**Conclusion:**

The results showed that a low level of emotional intelligence was associated with chronic migraine. The current results could help clinicians in planning for successful pain management/prevention programs.

## INTRODUCTION

Headache is one of the most common chronic pains. Evidence indicated that headache accounts for 3-4.5% of annual referrals to emergency wards in the U.S. ^1^ Based on the International Classification of Headache Disorders (ICHD), headaches are categorized into primary and secondary categories. ^2^ Primary headaches refer to those without any underlying diseases or medical reasons. In contrast, secondary headaches occur due to a prior disorder. ^3^ The most common and frequent primary headaches include migraine, tension type headache, and cluster type headache. Migraine is considered to be chronic in case the symptoms continue for 15 days a month for at least three months. ^4^

Migraine is the most prevalent type of chronic headaches. Based on the criteria of the International Headache Society, migraine is diagnosed as a recurrent, unilateral, and throbbing headache, which lasts for 4-72 hours, prevents physical activities, and is accompanied with nausea, vomiting and sensitivity towards light and noise. ^4^ One-year prevalence of migraine has been estimated 15% worldwide. ^5^ Also, the overall incidence of migraine was estimated 8·1 per 1000 person-years in individuals without migraine initially. ^5^ The prevalence of migraine in Iran, which is estimated 14%, is similar or even higher than that reported in some regions of the world. ^6^ Research has shown that psychological factors were effective in continuation and intensification of migraine. Therefore, researchers have investigated the factors related to headaches more seriously in the recent years. Based on the results, stress has been reported as one of the effective factors in the occurrence and frequency of migraine. Frequent stresses could result in migraine as well as psychological problems such as depression and anxiety. ^7^ In this context, identification of the variables that can influence the individuals’ stress levels is of paramount importance. ^8^

Emotional intelligence is among the concepts that have attracted a lot of attention in the recent years. Some studies have demonstrated that emotional intelligence could have an impact on individuals’ stress levels. ^9 , 10^ Individuals with higher emotional intelligence have been reported to possess better communication abilities, more efficient anger management abilities, and lower stress and anxiety levels. ^9^ One previous study found that emotional intelligence was significantly correlated with chronic pain in adolescents. ^11^ In contrast, the results of a recent study on adults showed that emotional intelligence did not have a significant effect on chronic migraine. ^12^ However, a recent study only compared chronic migraine to acute migraine and not to general healthy population. Therefore, any definite conclusion about the association between emotional intelligence and migraine needs more studies and investigations.

Anger is normally experienced as a transient natural emotion. However, in case of frequent arousal, it could cause a challenge and endanger people’s psychological adoptability and health. ^13^ Although improper expression of anger could hurt the surrounding people and objects, its suppression could hurt individuals both physically and mentally. This implies the importance of anger management. The undesirable effects of anger on individuals’ physical and mental statuses as well as on their interpersonal relationships have attracted the researchers’ attention to aggressive behaviors. ^14^ According to a recent study, 76.9% of Iranian adults have some degrees of anger and aggression. ^14^ Not controlling aggressive behaviors could lead to a variety of physical and mental problems, such as gastric ulcer, migraine, depression, and even suicide. ^15^ It has also been shown that anger is significantly higher in patients with chronic migraine compared to those with acute migraine. ^12^ Another study also found that anger symptoms were more common in migraine patients with aura compared to those without it. However, they did not include male patients and chronic migraineurs in their study, which might affect the results of their study. ^16^ Contrary to these findings, another study did not find a correlation between impulsivity and chronic migraine. They have discussed that the lack of a correlation between impulsivity and chronic migraine may be due to the use of an inappropriate screening tool in their study. ^17^

Based on what was mentioned above, emotional intelligence and intensity of anger may be effective in the occurrence or frequency of chronic migraine. However, up to now, there are few studies investigating the associations of emotional intelligence and anger with chronic migraine. ^12 , 16 , 17^ To the best of our knowledge, there has been no published original research on the comparison of migraine patients and healthy individuals regarding the emotional intelligence and anger. In addition, some previous studies included headache patients based only on the self-report rather than clinical diagnosis based on international consensus-based criteria, which might affect the quality of their findings. ^1 , 7^ Moreover, generalization of the results of some previous studies is difficult due to their target populations (e.g. including only female patients) and small sample size.^16 , 17^ Given the discrepancy in the study results, the fact that migraine is common, and due to widespread impacts of chronic migraine on daily life as well as high costs of migraine, it is necessary to identify psychosocial factors related with this type of pain. It has been strongly suggested that more research should identify psychosocial factors related with migraine, which may consequently improve pain management of these patients. ^1^ If associated factors for migraine can be identified, this can also provide a basis for preventive interventions. Hence, the present study aimed to make a comparison between migraine patients and healthy individuals in terms of emotional intelligence and anger. In addition, we investigated the predictive role of emotional intelligence and intensity of anger in chronic migraine. 

## MATERIALS AND METHODS

This comparative study was carried out on 494 individuals including patients with chronic migraine (n=250) and healthy controls (n=244) in Shiraz between August 2019 and February 2020. The sample size was calculated using the formula below using α=0.05, β=0.01, the mean difference of 2.5and standard deviations of 7.3, and S_2_ of 2.4, based on the results of a previous study. ^18^ By considering a 30% attrition rate, the final sample size for both groups was determined 452.

$n=\frac{{\left(Z_{1-\frac{\alpha }{2}}+Z_{1-\beta }\right)}^{2}(S_{1}^{2}+S_{2}^{2})}{{\left(\mu_{1}-\mu_{2}\right)}^{2}}$

$n=\frac{{\left(1.96+2.33\right)}^{2}(7.3^{2}+2.4^{2})}{{\left(2.5\right)}^{2}}$

The participants with chronic migraine were selected by convenience sampling. In doing so, patients with migraine (according to a pain specialist’s opinion based on the international Headache Society’s criteria) who had referred to morning and evening clinics affiliated to Shiraz University of Medical Sciences were invited to take part in the research. They were informed about the study objectives and their written informed consent forms were obtained. Afterwards, they were requested to complete the study questionnaires.

The healthy controls were selected by multistage sampling. In so doing, Shiraz was divided into four geographical districts, each of which being divided into four postal areas. Then, a park in each area was selected randomly (simple randomization). Parks were chosen because they are the locations where healthy people attend on a daily basis. In total, 16 parks were selected for data collection. In order for the sample to be a proper representative of the research population, 15-16 participants were selected from each park in the morning and evening. Then, the study objectives were explained to the participants and their written informed consent forms were obtained. Afterwards, the participants were asked to complete the questionnaires. Finally, the questionnaires were gathered, and data were entered into the system for analysis.

The inclusion criteria for the patients were being aged above 18 years, being willing to take part in the research, having at least the primary school degree, and suffering from primary chronic migraine based on a pain specialist’s diagnosis and the International Headache Society’s criteria. ^2^ The inclusion criteria for healthy people were age above 18 years, willingness to cooperate, subjects with at least the primary school degree, and self-reported physical and mental health. The exclusion criteria for the patients were suffering from other pains such as low back pain, having other chronic diseases such as diabetes and Multiple Sclerosis (MS), and suffering from chronic mental disorders such as depression based on self-report. Healthy individuals were excluded from the study if they expressed dissatisfaction with the use of their completed information.

In addition to the standard sociodemographic assessment (age, sex, marital status and educational level), the following variables were measured:

Emotional intelligence was assessed by the emotional intelligence appraisal^TM^ test. ^19^ This questionnaire is designed in five domains including general emotional intelligence and self-awareness (items 1-15), self-regulation (items 16-19), social awareness (items 20-27), and management of relationships (item 28) on a 6-point scale from 1 to 6. The total score ranges from 28 to 168. The scores >80 indicate a high level of emotional intelligence and those <60 show poor emotional intelligence. ^19^ The reliability of the instrument was confirmed with internal consistency, Cronbach’s alpha coefficient ranging from 0.85 to 0.91. Content validity was determined through expert development of items related to each of the subscales. After the face validity of the items was verified, subject matter experts eliminated unnecessary or repetitive items. Analysis of the construct validity of the emotional intelligence appraisal^TM^ suggests that the best fit for the model as presented in the assessment is an overall score with some division along the lines of personal and social competence. The principal component analysis suggested a two-factor solution, with a loose division between personal and social competence. These two factors accounted for 38.3% of the variance in the correlation matrix. ^19^ In a study by Ganji et al., the reliability (Cronbach’s alpha=0.83) and validity of the Persian version of the instrument were confirmed. The construct validity of the Persian version of the scale was assessed by correlating its score with the Bar-On emotional intelligence Questionnaire (r=0.67). ^20^

Anger was assessed by the Provocation Inventory, which is a 25-item self-report instrument measuring anger intensity. ^21^ It identifies five provocation situations including disrespectful treatment, unfairness/injustice, frustration/interruption, annoying traits, and irritations that induce anger in particular individuals. Participants were requested to answer the degree of anger in each situation according to a four-degree Likert scale (from “very little” to “very much”). The total score ranges from 0 to 100. Higher scores indicate greater anger. The internal reliability alpha for the PI was 0.95, ^21^ and the validity of the PI has been supported in a variety of samples. The convergent validity of the PI was confirmed by strong correlations found between the State-Trait Anger Expression Inventory subscales and Multidimensional Anger Inventory subscales with all five subscales of the PI. ^21^ The Persian version of the scale has been shown to be a reliable (Cronbach’s alpha=0.86) measure of anger. The construct validity of the Persian version of the scale was assessed by correlating its total score with other questionnaires that intended to measure anger such as Buss and Perry Aggression Questionnaire (r=0.78). Factor analysis revealed three factors, accounting for 39.4% of variances in anger scores. ^22^

The data were analyzed using SPSS software (version 22.0). The t-tests and Chi-square tests were used to compare the healthy individuals and patients with chronic migraine in terms of demographic and psychological variables, i.e. anger and emotional intelligence. The method of binary logistic regression assessed the association of every potential predictor (independent variable) with chronic migraine. Independent variables included age, sex, education level, marital status, anger, and emotional intelligence. The “Enter” method was used to enter variables into the analysis. Demographic variables were used as the baseline in the regression model. The significance level was set at P <0.05.

Ethical approval was obtained from the local Ethics Committee of Shiraz University of Medical Sciences (IR.SUMS.REC.1397.812). Eligible participants were informed about the study objective, and the voluntary nature of their participation. Written informed consent form was filled out by all participants. The data were collected anonymously without name lists.

## RESULTS

The calculated sample size was estimated to be 452. Nonetheless, to increase the precision of the results, the sample size was raised to 510 (255 subjects in each group). Of the 510 included subjects, 16 had to be excluded from the study because they experienced a chronic disease not related to pain (such as diabetes). Finally, 494 individuals remained in the study.

Based on the results presented in Table 1, there was no significant difference between the two groups regarding the participants’ mean age (P=0.05). Most of the participants in both groups were female 383 (77.5%) married 420 (85.0%) and had primary or secondary level of education 358 (72.4%). The results of chi-square tests revealed no significant relationships among migraine and sex (P=0.07), marital status (P=0.16), and education level (P=0.25).

**Table 1:** Sample description and analysis of group differences

Variables	Healthy group n=244	Chronic migraine n=250	T/X^2^	P value
	Mean±SD	Mean±SD		
Age	37.56±8.48	39.11±29.48	0.05*	0.05
	**N (%)**	**N (%)**		
Sex			2.39	0.07**
Female	182 (74.6)	201 (80.4)		
Male	62 (25.4)	49 (19.6)		
Marital status			5.05	0.16**
Single	38 (15.6)	32 (12.8)		
Married	202 (82.8)	218 (87.2)		
Divorced	3 (1.2)	0		
Widowed	1 (0. 4)	0		
Educational level			0.59	0.25**
Primary or secondary education	173 (71)	185 (74)		
University degree	71 (29)	65 (26)		

* T-test,

** Chi-square

The results of independent t-tests indicated that the mean intensity of anger was significantly higher among the patients with chronic migraine (51.52±15.66), compared to the healthy controls (28.39±9.85) (t (492)=19.69; P<0.001, Table 2). The results of independent t-test also showed that the mean of emotional intelligence was significantly lower among the patients with chronic migraine (75.92±8.23), in comparison to the healthy controls (116.23±12.28) (t (492)=42.74, P<0.001, Table 3).

**Table 2:** Comparison of psychological variables between the healthy group and patients with chronic migraine

Variables	Healthy group	Chronic migraine	T	P value
	Mean±SD	Mean±SD		
Anger	28.39±9.85	51.52±15.66	19.69	P<0.001 *
Emotional intelligence	116.23±12.28	75.92±8.23	42.74	P<0.001 *

* T-test

**Table 3:** Binary logistic regression: Slope coefficients, odds ratio (migraine/healthy group), 95% confidence intervals and significance

Predictors of migraine headaches	B^a^	OR^b^	CI^c^	P value*
Age	-0.02	0.97	0.84-1.12	0.72
Sex	0.94	0.39	0.009-16.25	0.62
Marital status	1.45	0.23	0.001-96.10	0.63
Education level	0.99	0.37	0.003-43.58	0.68
Anger	0.05	1.05	0.96-1.14	0.24
Emotional intelligence	-1.13	0.32	0.16-0.62	<0.001	

Reference group in regression analysis: healthy group,

* Logistic regression,

a Slope coefficient,

b Odds ratio (migraine/healthy group),

c Confidence interval

Binary logistic regression revealed that neither age (OR=0.97, CI=0.84-1.12; P=0.72), sex (OR=0.39, CI=0.009-16.25; P=0.62), marital status (OR=0.23, CI=0.001-96.1; P=0.63) and education level (OR=0.37, CI=0.003-43.58; P=0.68), nor anger (OR=1.05, CI=0.96-1.14; P=0.24) was significantly associated with chronic migraine. However, emotional intelligence (OR=0.32, CI=0.16-0.62; P<0.001) was significantly associated with chronic migraine.

## DISCUSSION

The present study found that the emotional intelligence in healthy adults was significantly higher than those with chronic migraine. The mean scores of anger intensity were significantly lower in healthy adults compared to patients with chronic migraine. Emotional intelligence, rather than anger intensity, was associated with chronic migraine after controlling for demographic characteristics. 

As expected, emotional intelligence was significantly lower among the patients with chronic migraine, and it could predict migraine headaches. In the same line, some researchers have shown that people with chronic pains have lower levels of emotional intelligence. ^11 , 12 , 23^ For instance, a recent study found that low emotional intelligence could play a critical role in the occurrence or frequency of chronic pains among adolescents. ^11^ Emotional intelligence refers to a set of verbal and non-verbal capabilities for identification and control of one’s feelings and emotions as well as those of others for better compatibility with the environment. It seems that there are various ways through which emotional intelligence could play a significant role in the occurrence or frequency of chronic pains. For example, emotional intelligence could help individuals to manage their emotions more efficiently. ^24^ It has been shown that individuals who accurately manage their emotions report fewer physical symptoms and less illness. ^25 , 26^ Moreover, there is some evidence that individuals with high emotional intelligence have greater self-efficacy in managing their pain. ^23 , 27^ Some research has also suggested that emotional intelligence might facilitate health outcomes through the use of different adaptive coping strategies and the ability to manage negative affect against stress. ^26 , 27^ The results of some studies have demonstrated that individuals with higher emotional intelligence were more satisfied with their lives, which could be attributed to their positive attitudes towards their daily lives as well as to their ability to perceive emotions more efficiently. ^28 , 29^ Also, higher emotional intelligence and better perception of one’s feelings and those of others are associated with reduced stress. ^30^ Stress is, in turn, an important factor associated with chronic pains. ^31^ Some researchers stated that high emotional intelligence could improve communication skills. ^32^ It has been shown that promotion of effective communication skills could result in a decrease in chronic pain. ^33^ Inversely, individuals with lower emotional intelligence showed more pain catastrophizing. ^24^ A significant relationship was also observed between low emotional intelligence and negative affectivity. ^27^ Pain catastrophizing and negative affectivity are, in turn, associated with increased experience of pain. ^34 - 36^

In the present study, the results of t-test indicated that the intensity of anger was higher among the migraine patients compared to the healthy controls. Anger and pain are negative emotions influencing each other through complex biological, affective, and behavioral mechanisms. ^26^ Anger can predispose one to, exacerbate, be a consequence of, or perpetuate pain. ^37 - 39^ Consistent with our findings, prior studies demonstrated the adverse effects of anger on chronic pain, treatment outcomes, and social relations. For example, some studies have revealed the impact of anger on reduction of pain threshold. ^37 - 39^ A recent study showed that anger expression styles, particularly anger-in, might affect the day-to-day adjustment of patients with chronic disease. ^37^ Another study reported that fibromyalgia patients showed a tendency to internalize and suppress anger. ^38^ Therefore, it seems that anger assessment and its management are important factors for an effective pain management and also for quality of life maintenance in chronic pain patients.

However, the results of regression showed that anger had no predictive effects regarding pain. This finding is inconsistent with the earlier results of some previous studies which revealed the association between anger and pain. ^16 , 37 , 38^ It should be noted that in multiple regression tests, variables share their predictive roles in the form of a regression model. ^40^ Therefore, entrance of anger and other variables, such as emotional intelligence, into the regression analysis might have caused anger to lose its predictive effect. Yet, further studies are recommended to be conducted on the issue.

In the current study, none of the demographic factors had predictive effects on migraine. This implied that psychological variables, such as emotional intelligence, played more important roles in prediction of migraine compared to age, sex, and education level. Nonetheless, future studies in this field are warranted.

The main strength of the present study was the inclusion of migraine patients based on clinical diagnosis according to the International Headache Society’s criteria, and it was not based on only self-report. Moreover, the present study included healthy controls to increase the validity of the findings. The large sample size is the other strength of the present study. 

Despite the promising implications of this study, a number of study limitations are worth noting. One of the limitations of the present study was that it was conducted on a sample of patients with chronic migraine referred to the clinics affiliated to Shiraz University of Medical Sciences who were selected via convenience sampling. This could affect the generalizability of the results. The study was a cross-sectional one; therefore, our findings do not elucidate a causal relationship. Further research with prospective design is needed. Another limitation of this study is related to assessing psychological variables based only on self-report questionnaires which might have negatively influenced our findings. Future studies are suggested to explore the predictive effects of other psychological variables on migraine headaches to help present a comprehensive program for helping patients with migraine. 

## CONCLUSION

Individuals who have lower levels of emotional intelligence might be more prone to chronic migraine. These findings indicate that emotional intelligence could play a role in the occurrence or frequency of chronic migraine. Although correlational results do not shed light on causal relationships, the present results add some evidence to further support the inﬂuence of psychological factors on chronic pain. Therefore, an effective preventive program for individuals at risk of chronic migraine should include a comprehensive psychological assessment. If these results are confirmed by interventional studies, interventions improving emotional intelligence are important in terms of preventing development of chronic migraine in adults. The current results could help clinicians in planning for successful pain management/prevention programs. In this context, holding training courses and developing appropriate therapeutic protocols can be effective.

## ACKNOWLEDGEMENT

The authors would like to thank all participants for their cooperation in the research. We would like to thank the Deputy of Research and Technology of Shiraz University of Medical Sciences for financial support (grant number: 17693).

**Conflict of Interest:** None declared.
